# Epidemiology of flavescence dorée and hazelnut decline in Slovenia: geographical distribution and genetic diversity of the associated 16SrV phytoplasmas

**DOI:** 10.3389/fpls.2023.1217425

**Published:** 2023-07-04

**Authors:** Zala Kogej Zwitter, Gabrijel Seljak, Tjaša Jakomin, Jakob Brodarič, Ana Vučurović, Sandra Pedemay, Pascal Salar, Sylvie Malembic-Maher, Xavier Foissac, Nataša Mehle

**Affiliations:** ^1^National Institute of Biology, Department of Biotechnology and Systems Biology, Ljubljana, Slovenia; ^2^Sensor Technologies, Jožef Stefan International Postgraduate School, Ljubljana, Slovenia; ^3^National Research Institute for Agriculture, Food and Environment, Fruit Biology and Pathology, Bordeaux, France; ^4^School for Viticulture and Enology, University of Nova Gorica, Nova Gorica, Slovenia

**Keywords:** epidemiology, plant disease, vectotype, *Vitis vinifera*, *Corylus avellana*

## Abstract

Flavescence dorée (FD) phytoplasma from 16SrV-C and -D subgroups cause severe damage to grapevines throughout Europe. This phytoplasma is transmitted from grapevine to grapevine by the sap-sucking leafhopper *Scaphoideus titanus*. European black alder and clematis serve as perennial plant reservoirs for 16SrV-C phytoplasma strains, and their host range has recently been extended to hazelnuts. In Slovenia, hazelnut orchards are declining due to 16SrV phytoplasma infections, where large populations of the non-autochthonous leafhopper *Orientus ishidae* have been observed. To better characterise the phytoplasma-induced decline of hazelnut and possible transmission fluxes between these orchards and grapevine, genetic diversity of 16SrV phytoplasmas in grapevine, hazelnut and leafhoppers was monitored from 2017 to 2022. The nucleotide sequence analysis was based on the *map* gene. The most prevalent *map* genotype in grapevine in all wine-growing regions of Slovenia was M54, which accounted for 84% of the 176 grapevines tested. Besides M54, other epidemic genotypes with lower frequency were M38 (6%), M51 (3%), M50 (2%) and M122 (1%). M38, M50 and M122 were also detected in infected cultivated hazelnuts and in specimens of *O. ishidae* leafhopper caught in declining hazelnut orchards. It suggests that this polyphagous vector could be responsible for phytoplasma infection in hazelnut orchards and possibly for some phytoplasma exchanges between hazelnuts and grapevine. We hereby describe new genotypes: M158 in grapevine as well as four never reported genotypes M159 to M162 in hazelnut. Of these four genotypes in hazelnut, one (M160) was also detected in *O. ishidae*. Analysis of additional genes of the new genotypes allowed us to assign them to the VmpA-III cluster, which corresponds to the 16SrV-C strains previously shown to be compatible with *S. titanus* transmission.

## 1 Introduction

Phytoplasmas, the wall-less plant pathogenic bacteria of the Mollicutes class, have a complex cycle (Lim and Sears, 1989; Marcone, 2014). They survive and multiply intracellularly in two different environments: the phloem sieve tube elements of the host plants and the body of the insect vector. Phytoplasmas are transmitted from plant to plant by hemipteran phloem feeders as leafhoppers, planthoppers, and psyllids (Weintraub and Beanland, 2006). They are associated with many plant diseases worldwide and can cause economic damage (Bertaccini et al., 2014). One of the quarantine diseases in the EU is caused by grapevine Flavescence dorée (FD) phytoplasma from taxonomic subgroups 16SrV-C and 16SrV-D (Davis and Dally, 2001). FD first occurred in southwestern France in the 1950s (Caudwell, 1957), and the disease continually spread to vineyards of southern European countries in the following decades (Tramontini et al., 2020). It was detected in Slovenia for the first time in 2005 (Mehle et al., 2011). The rapid epidemic spread of FD phytoplasma was caused by the vector *Scaphoideus titanus*, which probably arrived in Europe from North America in a single large introduction event (Papura et al., 2012). *S. titanus* is an efficient vector for propagating the FD phytoplasma in vineyards due to its specialisation for *Vitis* spp. and its ability to reach high population abundance (Chuche and Thiéry, 2014). Despite mandatory control measures, which consist of uprooting and destroying infected plants and spraying insecticides against *S. titanus*, FD phytoplasma continues to spread in Europe (Tramontini et al., 2020).

FD phytoplasma was not introduced in Europe simultaneously with *S. titanus*. Malembic-Maher et al. (2020) demonstrated that European alders (*Alnus* sp.) are the original reservoir for 16SrV-C phytoplasmas. The discoveries were made based on the sequence of methionine aminopeptidase (*map*) genetic locus, which shows higher variability in 16SrV phytoplasmas than the 16S rRNA gene. More than 150 different *map* genotypes were detected in wild alders and secondary host plants from the environment of vineyards all around Europe, but only 12 were associated with FD outbreaks on grapevine (*Vitis vinifera*) (Radonjić et al., 2013; Atanasova et al., 2014; Krstić et al., 2018; Plavec et al., 2019; Malembic-Maher et al., 2020; Krstić et al., 2022). The secondary hosts of 16SrV-C phytoplasmas include clematis (*Clematis vitalba)* (Angelini et al., 2004; Filippin et al., 2009; Casati et al., 2017; Plavec et al., 2019; Rossi et al., 2019), tree of heaven (*Ailanthus altissima)* (Filippin and De Pra, 2011; Miladinović et al., 2019; Plavec et al., 2019), Spanish broom (*Spartium junceum*) (Rizza et al., 2021) and willow (*Salix* spp.) (Casati et al., 2017). FD phytoplasmas are divided into three *map* genetic clusters: Map-FD1, Map-FD2, and Map-FD3 (Arnaud et al., 2007). Genotypes that do not belong to one of the three Map-FD clusters are not transmitted by *S. titanus* and do not provoke FD outbreaks (Malembic-Maher et al., 2020). Whether a phytoplasma variant is transmissible by *S. titanus* can be deduced from the *VmpA* sequence, a gene coding for a surface protein that functions as an adhesin and binds to insect vector cells (Arricau-Bouvery et al., 2021). Sequences of *VmpA* form three clusters, out of which clusters II and III are associated with *S. titanus* transmission and thus potential outbreaks of FD (Malembic-Maher et al., 2020). Up to now epidemic isolates from clusters Map-FD1, Map-FD2, and Map-FD3 were never found clustering in VmpA cluster I (Malembic-Maher et al., 2020).

In Slovenia, phytoplasmas of 16SrV-C subgroup have also been detected in decayed cultivated hazelnuts (*Corylus avellana*) (Mehle et al., 2019). Hazelnut is one of the ten most cultivated fruits in Slovenia and was intensively cultivated on an area of 190 ha in 2021 (Food and Agriculture Organisation of the United Nations, 2022). In some Slovenian hazelnut orchards, symptoms of phytoplasma infection were observed as yellowing and curling of leaves, dead branches leading to withering of entire bushes. These symptoms were linked to phytoplasmas of 16SrXII (‘*Candidatus* P. fragariae’), 16SrIX and 16SrV groups (Mehle et al., 2019). Molecular analysis of the FD9 marker and ribosomal protein operon sequences on 16SrV phytoplasma infected samples revealed the presence of FD-D and FD70 isolates, and sequencing of *map* gene classified them as Map-FD1 and Map-FD2 (Mehle et al., 2019). In addition, 16SrV phytoplasmas were detected on asymptomatic wild hazelnuts in the vicinity of FD infected vineyards in Switzerland (Casati et al., 2017). In this study, the presence of all three Map-FD clusters was evidenced in hazelnut shrubs as well as in the Asian leafhopper, *Orientus ishidae*, captured in the vicinity of vineyard (Casati et al., 2017).

In Slovenia, the 16SrV group phytoplasmas were detected for the first time in the Asian leafhopper *O. ishidae* in 2009 (Mehle et al., 2010). It is a polyphagous alien species that feeds mainly on broadleaf trees and shrubs (Lessio et al., 2016). Spatial distribution studies have shown that it is more abundant in host plants outside vineyards than in vineyards themselves (Lessio et al., 2016; Casati et al., 2017). Nevertheless, it can complete its life cycle on the grapevine (Desqué et al., 2019; Lessio et al., 2019) and it can transmit 16SrV phytoplasmas to grapevine after experimentally forced acquisition (Lessio et al., 2016). FD *map* genotypes transmitted by *O. ishidae* were demonstrated to be compatible with *S. titanus* transmission and therefore can potentially cause epidemics in grapevine (Malembic-Maher et al., 2020).

The objective of this study was to look at 16SrV phytoplasmas infected hazelnut orchards in Slovenia in order to determine which *map* genotypes might be the cause of their decline and whether these genotypes can be found also in potential vector *O. ishidae*. We compared the results with the structure of *map* genotypes in Slovenian FD infected vineyards. Newly detected genotypes were further genetically characterized to determine their possible origin and their epidemic potential on hazelnut and grapevine.

## 2 Materials and methods

### 2.1 Plant and insect material

**Figure 1:**
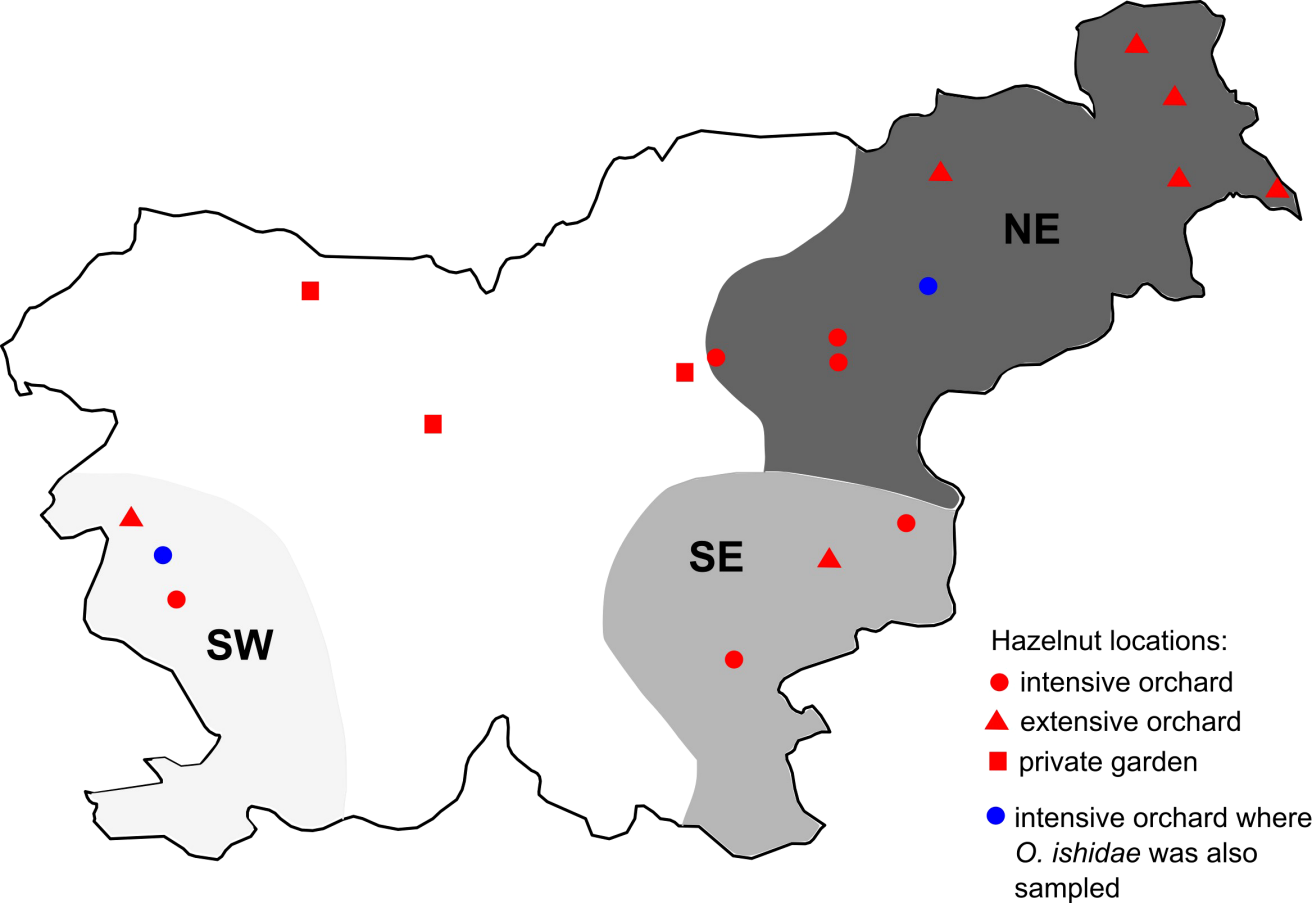
Map of grapevine, hazelnut, and leafhopper sampling regions/locations; Slovenian grapevine regions are shown in grey (NE in dark, SE in medium, and SW in light grey); locations of sampled hazelnuts are marked with dots (see legend); *S. titanus* samples were taken in NE and SE region.

Sampling plan is summarized in **Figure 1** and **Table 1**. Names and geographical origins of the genotyped samples are listed in **Supplementary Table 3**.

**Table 1:** 16SrV phytoplasma positive samples of grapevine, hazelnut, and leafhoppers from different regions of Slovenia between 2017 and 2022.

Region/host	Total number of samples*/number of 16SrV positive samples/number of samples with determined *map* sequence			
	Grapevine	Hazelnut	*O. ishidae*	*S. titanus*
NE	762 / 341 / 133	72** / 40** / 30**	48 / 8 / 6	15 / 7 / 5
NW	nt	2 / 1 / 0	nt	nt
SE	598 / 104 / 13	3 / 1 / 0	nt	6 / 0 / 0
SW	455 / 86 / 30	20 / 5 / 0	17 / 5 / 3	nt
total	1815 / 531 / 176	97 / 47 / 30	65 / 13 / 9	21 / 7 / 5

NE, northeast; NW, northwest; SE, southeast; SW, southwest; nt, not tested.

* Some samples of grapevine, *O. ishidae* and *S. titanus* were analysed as pools of up to five plants or up to seven leafhoppers.

** For some of these samples, roots and shoots were analysed separately.

The grapevine samples from symptomatic grapevines were collected as part of the official monitoring of grapevine yellows disease under the direction of Administration of the Republic of Slovenia for Food Safety, Veterinary and Plant Protection. This study includes grapevine samples collected between 2017 and 2021 in 107 different sites in three different wine-growing regions of Slovenia: the northeast (NE), the southeast (SE) and the southwest (SW). Each sample consisted of leaves from at least three different parts of one or up to five plants from the same vineyard.

Symptomatic hazelnut samples were collected as part of a survey for phytoplasmas in Slovenian hazelnut orchards (seven extensive and eight intensive) and in three private gardens. They were collected from 2017 to 2021, from the end of June to the end of October. The sample consisted of leaves from three different parts of a decaying shrub. In most cases, samples were also taken from roots - excavated from three different parts of the shrub rooting system and treated as a separate subsample.

Leaf samples of grapevine and hazelnut were prepared in the same manner by excising 1 g of leaf mid-vein tissue. Hazelnut roots were washed extensively to remove all soil particles, and then 1 g of phloem tissue was excised. All samples were stored at -20°C until further analysis.

Leafhoppers were captured by sweep net within vineyards or hazelnut orchards during the period when plant symptoms were expressed: *S. titanus* between August and September in years 2021 and 2022 and *O. ishidae* in July 2019 and 2021. *S. titanus* were captured in the frame of the national monitoring in five different vineyards in NE region and stored in 70% ethanol until analysis. *O. ishidae* was captured in two hazelnut orchards infected with 16SrV phytoplasmas (Črešnjevec and Šempas) and immediately stored at -80°C or in 70% ethanol.

### 2.2 DNA extraction and phytoplasma detection

After homogenization with Plant DNA lysis buffer (QuickPick Plant DNA Kit; Bio-Nobile, Finland) or ELISA buffer (Kogovšek et al., 2015) of 1 g of leaf veins or roots using a FastPrep24™ instrument (MP Biomedicals, Illkirch-Graenstaden, France), total DNA was extracted using the QuickPick™ SML Plant DNA Kit (Bio-Nobile, Finland) and a KingFisher extraction device (ThermoScientific, USA) (Mehle et al., 2013a).

Leafhoppers were homogenised individually or in groups of two to seven specimens. In total 77 *S. titanus* specimen (analysed as 21 samples) and 105 *O. ishidae* (analysed as 65 samples) were tested. In case of storage in ethanol, they were rinsed in four consecutive water baths before homogenization. The leafhoppers were homogenised in a 1.5 ml tube with liquid nitrogen and a sterile plastic pestle. Total DNA was isolated following the same protocol as for plant material, with some adjustments: if <3 specimen was in sample, volume of lysis buffer used was 400 μl and DNA was eluted in 100 μl or if ≥3 specimen was in sample, volume of lysis buffer was 600 μl and DNA was eluted in 200 μl.

Tenfold diluted total plant or insect DNA (except undiluted insect DNA in case of one specimen per sample) were tested using a specific real-time PCR for 16SrV phytoplasma group (Hren et al., 2007). The setup of the real-time PCR was as described in (Mehle et al., 2013b). All samples were analysed also with universal phytoplasma real-time PCR assay (Christensen et al., 2004) and grapevine samples additionally with specific real-time PCR assay for Bois noir phytoplasma (Hren et al., 2007). In addition to the positive and negative amplification control in each real-time PCR run, the 18S rRNA assay (Applied Biosystems, Massachusetts, USA) was used as a control of DNA extraction.

### 2.3 Amplification and sequencing of genetic markers

Samples which tested positive with 16SrV phytoplasma-specific real-time PCR assay and whose Cq value was less than 32 were subjected to further amplification of the *secY-map* locus (*map* gene) by nested PCR (nPCR) with FD9f5/MAPr1 and FD9f6/MAPr2 primer pairs (Arnaud et al., 2007). First and second PCR reactions were performed in 50 μl with 1x High fidelity PCR Buffer (Invitrogen, Massachusetts, USA), 2mM MgSO_4_, 0.2 mM dNTP, 0.2 μM of each primer and 1U of Platinum Taq DNA Polymerase High Fidelity (Invitrogen, Massachusetts, USA). 2 μl of 10-fold diluted plant or insect DNA (except in case of one specimen per sample where it was undiluted) was used as a template in the first PCR and 2 μl of 100-fold diluted PCR product was used for the second PCR. The conditions were the same for both PCR reactions: initial denaturation at 94°C for 2 min; 35 cycles consisting of denaturation at 94°C for 15 s, annealing at 52°C for 30 s and extension at 68°C for 1 min. Each run included negative template amplification control (water). Amplification of nPCR was checked by 1% agarose gel electrophoresis with ethidium bromide (EtBr) staining and visualisation under UV illumination. Nested PCR products were purified (outsourced to Macrogen Europe commercial service or with MinElute PCR Purification Kit (Qiagen, Hilden, Germany) and forward and reverse Sanger sequencing was performed by Macrogen Europe (Amsterdam, the Netherlands) or Eurofins GATC (Ebersberg, Germany).

Samples, in which new *map* genotypes were discovered, were further characterised with nPCR and Sanger sequencing of five different genes: *tuf*, *rplV*, *rplF, dnaK* and the first repeat of *vmpA* (*vmpA-R1*, information on tests in **Supplementary Table 1**). Primer sets for *tuf, rplF* and *dnaK* were changed compared to previous studies to improve sequence quality (**Supplementary Table 1**). PCR reactions were performed with *Taq* DNA Polymerase with Standard *Taq* Buffer (NEB, Massachusetts, USA), 3 mM final concentration of MgCl_2_ and 5% DMSO in the first PCR reaction. Amplification of nPCR was checked by 1% agarose gel electrophoresis with EtBr staining and visualisation under UV illumination. Nested PCR products were purified and sequenced with Macrogen Europe (Amsterdam, the Netherlands).

### 2.4 Sequence analysis

Forward and reverse (or both forward for *VmpA-R1)* raw chromatograms of all genes were assembled, checked for quality and discrepancies, and trimmed in CLC Genomic Workbench (version 21.0.1, Qiagen) or with the Staden package (Bonfield et al., 1995). Contigs were compared to the local databases of various genes using BLAST algorithms (http://www.ncbi.nlm.nih.gov/blast). Location map and unrooted median-joining networks of *map* genotypes were generated using PopArt 1.7 software (Leigh and Bryant, 2015) with median-joining method and parameter e = 0 on alignment made with ClustalW in MEGA7 (Kumar et al., 2016). Sequence alignments of *VmpA*-*R1*, *dnaK* and multilocus sequence analysis (MLSA) of concatenated *tuf* (925 bp) – *rplV* (795 bp) – *rplF* (807 bp) – *map* (674 bp) were also performed using ClustalW in MEGA7. The phylogenetic trees were constructed using Maximum Parsimony algorithm in MEGA7. The statistical support of the branches was evaluated using the bootstrap method based on 500 replicates. All the accession numbers of the different genes from different isolates are listed in **Supplementary Table 2** and in (Malembic-Maher et al., 2011) for the MLSA.

## 3 Results

### 3.1 Grapevine and hazelnut infection with 16SrV phytoplasmas

A total of 1815 symptomatic grapevine samples were sampled between 2017 and 2021 in three wine-growing regions of Slovenia (**Table 1**). The proportion of 16SrV phytoplasma infected grapevine samples increased over time, and was the highest in 2021 (49%, 169 out of 347 samples analysed). In 2021, there was a large outbreak in NE region and it was more extensively sampled. The five-year overall percentage of 16SrV positive samples was 29%. In each of the three wine-growing regions, 4-6% of 16SrV-positive samples were also infected with phytoplasma Bois noir (16SrXII, ‘*Ca.* Phytoplasma solani’). During this five-year period, 50% of all sampled grapevines were infected with Bois noir phytoplasma and 21% were phytoplasma negative.

From 2017 until 2021, a total of 97 hazelnut shrubs from 18 different locations were analysed. The 16SrV phytoplasmas were detected in 47 shrubs (**Table 1**). ‘*Ca*. P. fragariae’ was also detected in some of the orchards and in one sample from a private garden in 2017 (Mehle et al., 2019), but no phytoplasma was detected in 41% of the sampled shrubs (tested with the universal phytoplasma assay, data not shown). 16SrV positive shrubs were found in intensive orchards (6 of 8 sampled), extensive orchards (3 of 7 sampled), and in one out of three private gardens. In one orchard monitored throughout the five-year period (Črešnjevec), the proportion of 16SrV-positive tests of decaying shrubs increased from 30% in 2017 (3 out of 10 shrubs) to 90% in 2021 (9 out of 10 shrubs). All 16SrV phytoplasma-positive shrubs showed dieback symptoms - most commonly wilting of individual branches, yellowing of leaves, or withering of the entire shrub. The real-time PCR analyses of shoot and root subsamples, which were taken from the same shrub, showed that the phytoplasma concentration was higher in the roots (data not shown).

### 3.2 Leafhoppers infection with 16SrV phytoplasmas

FD phytoplasmas occurrence was analysed in 21 cumulative *S. titanus* samples (up to seven specimens per sample) collected between August and September 2021 and 2022. Using the real-time PCR assay of Hren et al. (2007) the presence of 16SrV phytoplasmas was detected in two samples of *S. titanus* in 2021 and five in 2022 - all caught in FD-infected vineyards in NE Slovenia.

In July 2019 and 2021, two hazelnut orchards infected with 16SrV phytoplasmas were subjected to insect vector searches - one in NE (Črešnjevec) and one in SW Slovenia (Šempas). *S. titanus* was not found in any of these hazelnut orchards. However, in 2021, the populations of *O. ishidae* in Črešnjevec and Šempas were estimated to be abundant, as 44 and 56 specimens were caught in one hour, respectively. In the NE orchard, 16SrV phytoplasmas were detected in two samples from 2019 (pools of four and two specimens) and in six samples composed of only one specimen in 2021. *O. ishidae* from the SW orchard were pooled in 17 pools of two to five specimens, and five pools tested positive for 16SrV phytoplasmas (**Table 1**).

### 3.3 *Map* sequencing of infected samples

The *map* gene of 16SrV phytoplasmas was sequenced in 176 grapevines and 30 hazelnuts, 11 of which were tested as separate subsamples of shoots (leaf veins) and roots (**Table 1**, details in **Supplementary Table 3**). Other samples had Cq value of 16SrV specific real-time PCR higher than 32, making the concentration of phytoplasma too low to submit for nPCR and Sanger sequencing. Out of 20 positive samples of leafhoppers, 14 samples were subjected to *map* sequencing: five were *S. titanus* (one sample of seven imagos and others of three imagos) and nine were *O. ishidae* (four single imago samples and five cumulative). Their Cq values ranged from 18 to 32 for the specific 16SrV phytoplasma assay. In total, 231 samples were submitted to *map* sequencing (**Supplementary Table 3**) with 2-fold coverage (except for nine samples, of which only one sequence was of sufficient quality). Single infections of a particular *map* genotype were identified in all samples.

**Figure 2:**
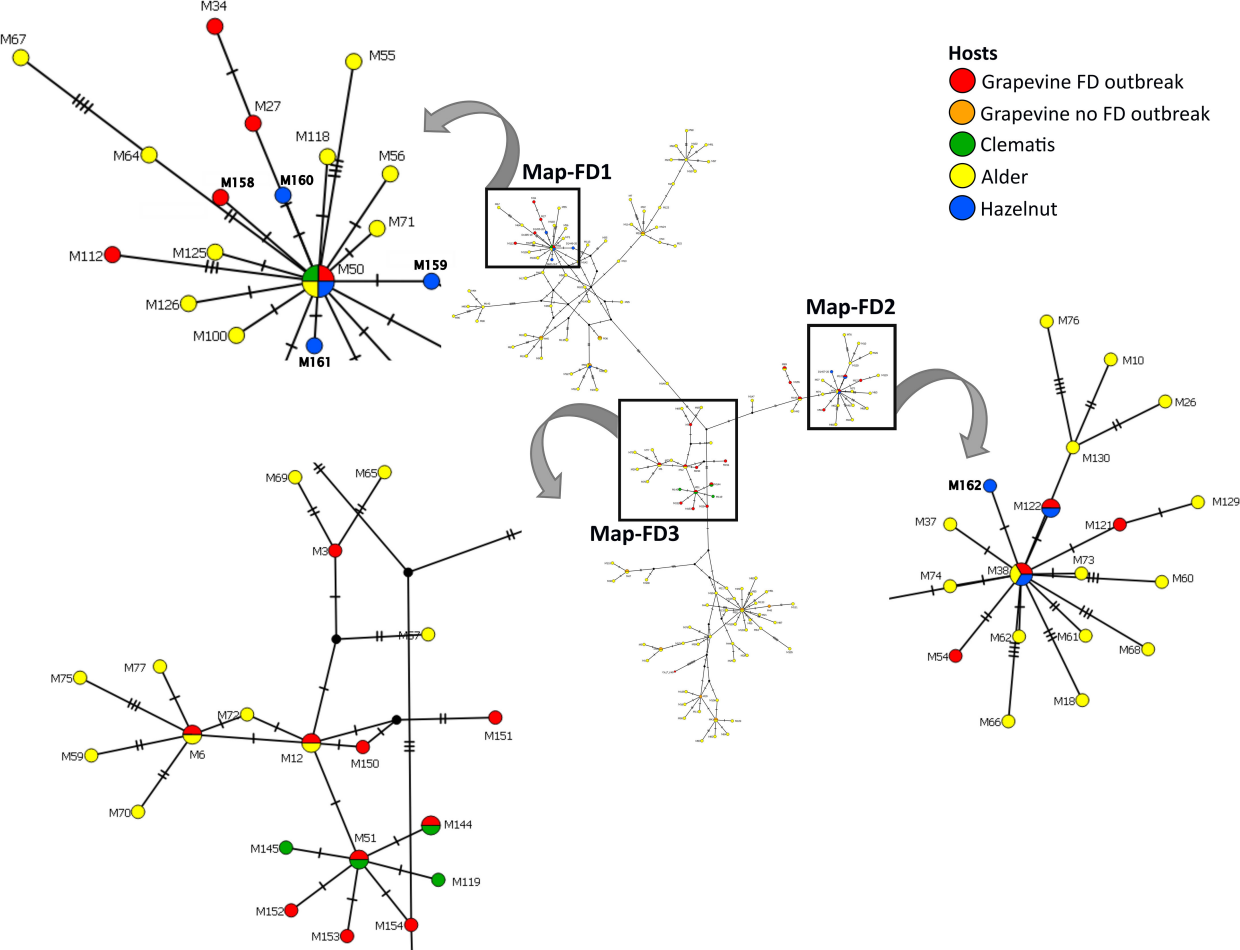
Median joining network of *map* genotypes (674 long amplicons); circles represent genotypes (paired genotypes are connected), black circles represent missing haplotypes and hatch mark represent one SNP; the legend shows color code of different hosts (in case of grapevine separated on genotypes causing FD and the ones not-causing FD outbreaks); squares mark Map-FD clusters that are enlarged; in the enlarged area new genotypes found in Slovenia are written in bold.

Eleven different *map* genotypes were characterized, of which five, M158 to M162, are new (**Table 2**). In a phylogenetic analysis, they were compared with reference sequences derived from epidemic FD strains, non-epidemic 16SrV-C isolates on grapevine (Palatinate Grapevine Yellows), and some 16SrV-C isolates from other plants described in previous studies (Malembic-Maher et al., 2020; Krstić et al., 2022) (**Figure 2**, **Table 2**). Four new genotypes (M158, M159, M160 and M161) group in the Map-FD1 cluster, which is the most variable in Slovenia. They are 99.85% identical to M50 with one nucleotide difference in the amplicon. A new *map* genotype found in three hazelnut shrubs (M162) belongs to Map-FD2 and differs from M38 by one nucleotide. The single nucleotide polymorphisms (SNP) in all newly discovered isolates represent transitions. The Map-FD3 cluster was the most homogeneous, as all *map* sequences were 100% identical to M51 (**Figure 2**; **Table 2**).

**Table 2:** Summary of discovered *map* genotypes from 2017 to 2022 in different hosts.

Map-FD cluster	Genotype	GenBank acc no.	Reference	Host	Number of samples
FD1	M50	AM384887	(Arnaud et al., 2007)	Grapevine	4
				Hazelnut	8*
				*O. ishidae*	1
	M158	OX417123**	This study	Grapevine	7
	M159	OX417124**	This study	Hazelnut	1
	M160	OX417125**	This study	Hazelnut	1
				*O. ishidae*	1
	M161	OX417126**	This study	Hazelnut	1*
FD2	M54	AM384886	(Arnaud et al., 2007)	Grapevine	147
				*S. titanus*	5
	M38	LN850369	(Elek et al., unpublished)	Grapevine	11
				Hazelnut	12*
				*O. ishidae*	5
	M122	LN850372	(Elek et al., unpublished)	Grapevine	2
				Hazelnut	2*
				*O. ishidae*	2
	M162	OX417127**	This study	Hazelnut	3*
FD3	M51	FN811141	(Malembic-Maher et al., 2011)	Grapevine	5
Grapevine non-epidemic isolate	M48	AM384893	(Arnaud et al., 2007)	Hazelnut	2*

* In 11 samples, roots and shoots were analysed separately (counted as one sample here; results of separate analysis are shown in **Supplementary Table 3**).

** sequences were deposited to European Nucleotide Archive (ENA) under https://www.ebi.ac.uk/ena/browser/view/OX417123-OX417147.

**Figure 3:**
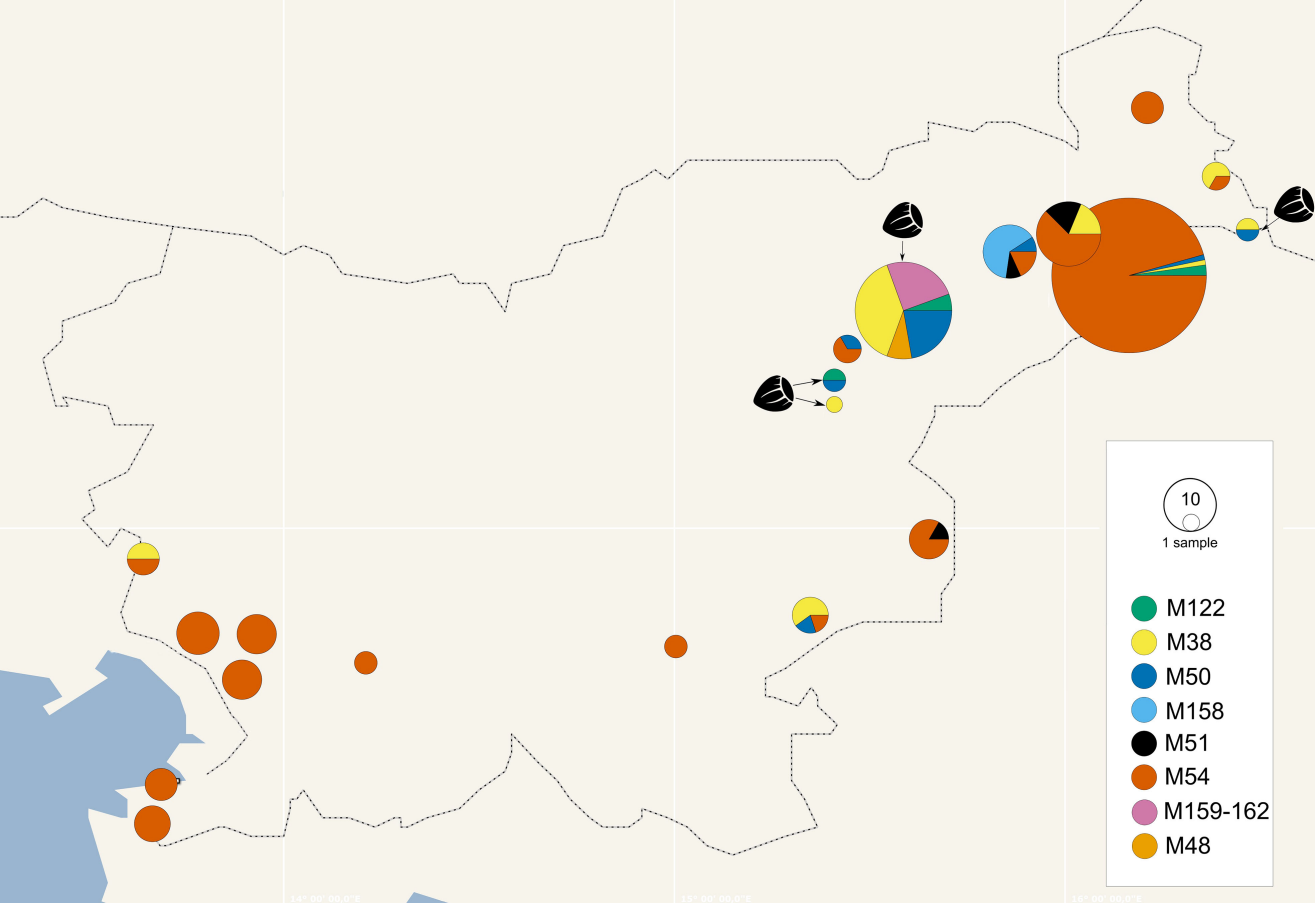
Geographical distribution of *map* genotypes in grapevines and hazelnuts (hazelnut samples marked with hazelnut image and arrow, other pie charts represent grapevine) collected in Slovenia from 2017 to 2021; the size of the circle corresponds to the number of samples and the colours to the *map* genotypes (see legend); where vineyards were close together, samples were combined in the same pie chart.

The *map* genotypes were determined on grapevine samples from all wine-growing regions of Slovenia: NE, SE, and SW. Six different genotypes were detected from all three Map-FD clusters (**Table 2**; **Figure 2**). The most prevalent genotype was M54 from Map-FD2, which was present in 84% of the sequenced samples. This genotype was also detected in all five samples of *S. titanus*. The other genotypes were much less abundant: M50 (2%) and M158 (4%) from Map-FD1, M38 (6%) and M122 (1%) from Map-FD2, and M51 (3%) from Map-FD3 (**Table 2**; **Supplementary Figure 1**). Genotypes M54, M51, and M158 were specific to grapevine. Interestingly, all six different genotypes were detected in NE, four in SE and only two in SW (**Figure 3**). The Map-FD2 genotypes M54 and M38 occurred in all wine-growing regions. They were the only ones present in SW with 28 samples of M54 in 16 different sites and two samples of M38 found at the same site in 2019 (Gradišče and Prvačino, **Supplementary Table 3**). Genotype M122 was detected only in NE, in two nearby sites (Stara Cesta and Žerovinci, **Supplementary Table 3**). The newly described M158 was also detected only in NE in seven samples from 2017 to 2019 at five nearby sites. Genotypes M50 and M51 were found in NE and SE. In NE, M50 was present in three different sites: at two it was the only genotype, but at one two other genotypes were detected in different years (M158 in 2017, M50 in 2019 and M54 in 2021). Interestingly, in every site harbouring more than one genotype, M54 was always detected except in Tolsti vrh, where it was M38 and M50 only. Up to three genotypes could be detected in a same site.

The hazelnuts infected with 16SrV phytoplasmas further characterized on *map*, all came from four orchards in NE Slovenia (**Figure 3**; **Supplementary Table 3**). Eight different genotypes were detected (**Table 2**; **Figure 3**). They grouped into two clusters - Map-FD1 and FD2, but none of the isolates belonged to Map-FD3 cluster. Six hazelnut shrubs (20%) were infected with *map* genotypes (M159-M162), which have not been described previously (**Table 2**; **Supplementary Figure 2**). These new genotypes were all found in an intensive orchard (Črešnjevec) in different years: M159 and M160 in root samples from 2020 (isolates D1448-20 and D1455-20), M161 in root and shoot samples of the same shrub from 2021 (isolate SB21-5-P), and M162 in root sample from 2020 and two shrubs (root and shoot samples) from 2021 (isolate SB21-10-K). In this orchard, all the eight genotypes described in hazelnut were present in the years from 2017 to 2021. Of 30 hazelnut shrubs analysed, 22 were infected with the same genotype as grapevine: M38 (40%), M50 (27%), and M122 (7%). Interestingly, the most prevalent genotype in grapevine M54 was not detected in hazelnuts. The analysed hazelnut samples were located in the same NE geographic area as grapevines infected with the same genotypes. In addition, hazelnut was found to harbour genotype M48 (7%), which is not part of the Map-FD clusters and has been previously described in Palatinate Grapevine Yellows cases (non-epidemic in grapevine) and in alders (Malembic-Maher et al., 2020). Eleven hazelnut shrubs that were analysed in subsamples had the same genotype in shoots and roots (**Supplementary Table 3**).

*O. ishidae* collected on hazelnuts harboured three genotypes of 16SrV phytoplasmas that are present in both hazelnuts and grapevines: M38, M50, and M122 (**Table 2**). In the SW orchard (Šempas), only one genotype was detected in *O. ishidae* (M38), while in the NE orchard (Črešnjevec), four different genotypes were detected (**Supplementary Table 3**). In addition to the three genotypes listed, the new genotype M160 was also detected in one pooled sample. Interestingly, one year later, in 2020, it was also detected in hazelnuts in the same orchard.

### 3.4 Finer genetic characterisation of new *map* genotypes

**Figure 4:**
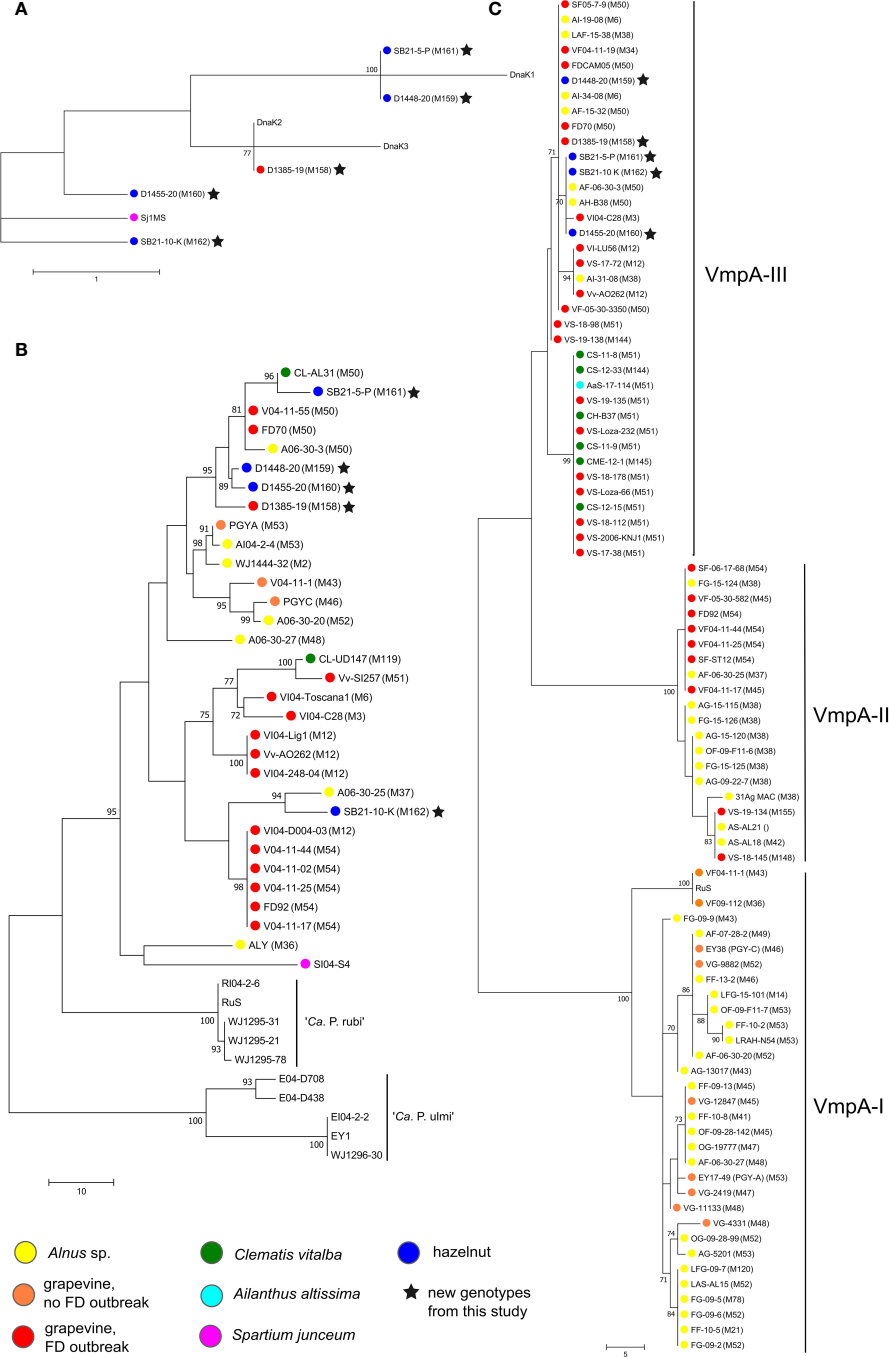
Unrooted phylogenetic trees constructed by maximum parsimony in MEGA7 software based on **(A)** *dnaK* (492 nt), **(B)** MLSA of concatenated *tuf* (925 bp) – *rplV* (795 bp) – *rplF* (807 bp) – *map* (674 bp) and **(C)** one of the trees constructed based on *VmpA-R1* (312 nt); sequences were aligned by ClustalW and number on branches are bootstrap values obtained from 500 replicates and indicated when ≥70%; the bar represents number of nucleotide changes; color circles represent different hosts (see legend); accession numbers are listed in **Supplementary Table 2** and in (Malembic-Maher et al., 2011).

The five new genotypes M158 to M162 were characterized more precisely by sequencing genetic markers *vmpA-R1, dnaK, tuf, rplF* and *rplV*. The results are shown in **Figure 4**. Based on the *dnaK* gene amplicon (475 bp), D1385-19 (Map-FD1, M158) detected in grapevine has the same sequence as the DnaK2 reference isolate collected in grapevine in northeast Italy (reference strain FD-C, MH547711) (**Figure 4A**). In the MLSA of concatenated *tuf* (925 bp), *rplV* (795 bp), *rplF* (807 bp), and *map* (674 bp) gene loci (**Figure 4B**), it is also clustered with Map-FD1 isolates collected in grapevine, clematis, and alder in northeast Italy and southwest France (with reference strain FD70 from grapevine). Analysis of the first *VmpA* repeat (VmpA-R1) showed that it belongs to the VmpA-III cluster that groups FD isolates transmitted by *S. titanus* (**Figure 4C**).

The four new *map* genotypes discovered in hazelnuts (M159-M162) differ from the reference sequences of *dnaK* (**Figure 4A**). M162 (isolate SB21-10-K) is most similar to *dnaK* from *Spartium junceum* (acc. no. MT629775, Sj1MS) with two nucleotide differences. M159 (isolate D1448-20) and M161 (isolate SB21-5-P) have one nucleotide difference from DnaK1 at the same nucleotide position towards the end of sequence and M160 (isolate D1455-20) one nucleotide difference from DnaK2 in the middle of the sequence. In the MLSA analysis, the isolates M159-161 group in a cluster with the greatest host diversity, which includes isolates from Map-FD1 along with the grapevine isolate D1385-19. Isolate SB21-10-K (genotype M162) is genetically related to isolates from Map-FD2 cluster (reference isolate FD92 from grapevine) and more closely related to an isolate collected in alder in southwest France. Interestingly, all hazelnut isolates also belong to the VmpA-III cluster gathering isolates compatible with *S. titanus* transmission (**Figure 4C**).

## 4 Discussion

FD disease has been present in Slovenia for more than a decade, and despite control measures, new outbreaks continue to occur. It causes damage and yield losses in all wine-growing regions of Slovenia. However, grapevine is not the only host of concern for 16SrV phytoplasmas as they were detected in several hazelnut orchards in Slovenia, where they caused severe damage to the production (Mehle et al., 2019). Our investigation of different *map* genotypes of 16SrV phytoplasmas present in grapevine, hazelnut, and the leafhopper vectors *O. ishidae* and *S. titanus* over a period of six years (2017-2022) in Slovenia shows that eleven *map* genotypes are present and of these, three are common to grapevine and hazelnut (M38, M50 and M122). Others were specifically detected only in grapevine (M51, M54, M158) or only in hazelnut (M48, M159-M162).

As we know from previous studies of FD phytoplasmas molecular diversity of Slovenian grapevine strains, they belong to three FD clusters based on nucleotide sequence and restriction fragment length polymorphism analysis of FD9 marker (Mehle et al., 2011). In this study, we went beyond and monitored *map* genotypes. Most FD outbreaks in Slovenian vineyards are associated with genotype M54 from Map-FD2 cluster. This genotype is the most abundant in France and in Slovenian neighbouring countries Italy, and Croatia (Plavec et al., 2019; Rossi et al., 2019; Malembic-Maher et al., 2020; Rizzoli et al., 2021). In Croatia, in the region of Istra near the Slovenian SW wine-growing region, Map-FD2 was the only genetic cluster discovered, and the high number of infected *S. titanus* in Istra suggests a high transmission efficiency (Plavec et al., 2019). M54, the only *map* genotype of the 16SrV-D taxonomic subgroup, was the only genotype detected in our *S. titanus* samples. Upon a broad continental survey, M54 was never found in alder trees, clematis, and alder leafhoppers (Malembic-Maher et al., 2020). In our study, M54 was also absent from hazelnut and *O. ishidae.* So, like in other European vineyards, this genotype seems restricted and adapted to the grapevine-*S. titanus* pathosystem, with a high epidemic potential. However, a study from Switzerland has shown that M54 can also occur in hazelnuts in the vicinity of vineyards infected with FD phytoplasma M54 genotype, and we cannot exclude a possible transmission to the wild compartment (Casati et al., 2017).

The other *map* genotypes present in Slovenian grapevines were much less frequent. The newly described M158, belonging to Map-FD1 cluster, was found specific to grapevine but present at five sites in the NE region in three consecutive years. Our MLSA analyses showed it was clustering together with isolates detected in alder, clematis, and grapevine, which might indicate a transfer from wild plants to grapevine or an evolution from a strain present on grapevine. Based on *VmpA-R1* sequence analysis, it belongs to the VmpA-III cluster, which means that it is compatible with *S. titanus* transmission and has epidemic potential on grapevine. The only Map-FD3 genotype detected in grapevine in Slovenia was M51. M51 whose natural non-vitis host is clematis, is the dominant genotype causing epidemics on grapevine in Serbia (Krstić et al., 2022). In Croatia its finding was restricted to the borders of Slovenia and Serbia (Plavec et al., 2019). M51 was also found in other hosts, *C. vitalba* or *A. altissima*, in Italy, Croatia, Hungary and Serbia (Plavec et al., 2019; Rossi et al., 2019; Malembic-Maher et al., 2020; Krstić et al., 2022). In Slovenia, clematis samples were previously analysed on the DNA segment of the FD9 marker, and they also belonged to the FD3 strains (Mehle et al., 2011). Thus, the M51 cases in Slovenia could either originate from locally infected clematis or be due to a transport of infected *S. titanus* or grapevine material from the neighbouring infected areas in Croatia.

In this study we detected three *map* genotypes that were common to grapevine and hazelnut (M38, M50 and M122). All three were previously found in the *S. titanus*-grapevine pathosystem causing possible outbreaks (Plavec et al., 2019; Malembic-Maher et al., 2020). A two SNP distant genotype of M54 is M38, which has been previously detected in alder and grapevine, is thought to be an ancestral genotype of M54 (Malembic-Maher et al., 2020). We observed the presence of M38 in all Slovenian wine-growing regions and in samples of hazelnut. The other genotype M122 detected in both hosts, grapevine and hazelnut, also belongs to the Map-FD2 cluster. M122, which differs from M38 by one SNP, was previously found in France, Hungary, and Croatia (Plavec et al., 2019). In addition to M38, M50 from Map-FD1 cluster is a genotype that appears to have escaped from alders. It is successful in the grapevine*-S. titanus* pathosystem (Malembic-Maher et al., 2020) and was here detected in 2% of Slovenian grapevine samples and 27% among hazelnut samples. Interestingly, M38, M50 and M122 genotypes from grapevine were also detected in hazelnuts sampled in the same large NE region, suggesting that phytoplasma exchanges could have occurred between both species, probably mediated by insect vectors. However, the locations of hazelnut orchards, where we found genotypes detected also in grapevine, are relatively far away from FD infected vineyards. The closest vineyard infected with M50 was 3.5 km of air distance away from hazelnut orchard where we also found M50. Based on these data, we could not determine a direct link between the 16SrV phytoplasmas in grapevine and in hazelnut. Other woody hosts between orchards and vineyards could also serve as a source of these genotypes, such as alder, which is a common original host of M38 and M50 in Europe (Malembic-Maher et al., 2020) and hazelnut might be, like grapevine, a secondary host. *Map* genotypes had previously been studied in Slovenia in only two hazelnut shrubs – one belonged to Map-FD1 and one to Map-FD2 (Mehle et al., 2019). In this study, where we analysed 30 hazelnuts, we also detected only genotypes from Map-FD1 and FD2 clusters. However, phytoplasmas of the 16SrV group were identified in asymptomatic, non-cultivated hazelnut bushes near FD-infected vineyards in Switzerland (Casati et al., 2017). There they detected genotypes from all three *map* clusters. In future studies, wild hazelnuts in Slovenia will be subjected to a phytoplasma search to determine whether they are infected and, if so, which genotypes are present in hazelnuts in the environment.

In a broad search for present *map* genotypes in Slovenia, we found one new genotype in grapevine (M158) and four new ones in hazelnut (M159-162). To better understand about their origin and their epidemic potential, we performed some additional analyses. Whether the genotype detected is compatible with transmission with *S. titanus* can be determined based on the *VmpA-R1* sequence, which codes for a protein important for adhesion to insect vector cells (Arricau-Bouvery et al., 2021). The three distinct clusters divide isolates into those that are transmissible by *S. titanus* (vectotype II and III) and those that are not (vectotype I) (Malembic-Maher et al., 2020). All new *map* genotypes discovered in this study, regardless of host (grapevine/hazelnut), belonged to vectotype III cluster, which groups isolates from the Map-FD1 and Map-FD3 clusters found in clematis, alder, and grapevine outbreaks (Malembic-Maher et al., 2020). In the same study, it was also shown that some isolates transmitted by *O. ishidae* belong to vectotype III cluster and that they are compatible with *S. titanus* transmission. Based on the genetic marker *dnaK* (Rossi et al., 2019), the newly discovered genotypes belong to two clusters - DnaK1 and DnaK2. One of the hazelnut samples - isolate SB21-10-K - was separated from the DnaK2 cluster along with an isolate from *Spartium junceum*. It is also the only isolate of the newly discovered *map* genotypes that clustered in the MLSA together with the reference strain FD92 and not with FD70 as the other four. The MLSA was based on four genes (*tuf, rplV, rplF, map*), but it would be better to look for genes throughout the whole genome in the future.

Regarding the spread of the disease, it is of outmost importance to also look for insect vectors in diseased hazelnut orchards. *O. ishidae* is an invasive species that has been efficiently expanding its territory since its first detection in the EU in 1998 in northern Italy, probably due to its wide range of feeding preferences, from woody to herbaceous plants (Guglielmino, 2005). Since invasive Hemiptera species are known to have an important impact on the spread of phytoplasmas, *O. ishidae* came into focus as a possible vector of FD (Mehle et al., 2010; Lessio et al., 2016; Casati et al., 2017; Lessio et al., 2019). Populations of *O. ishidae* in the two hazelnut orchards studied in 2021 were numerous and the insects harboured 16SrV phytoplasmas. High populations of *O. ishidae* were also observed in Switzerland, in a forest adjacent to a FD-infected vineyard and composed mainly of hazel and willow (Casati et al., 2017). The genotypes detected in this study in *O.ishidae* were the same as those found in hazelnuts: M38, M50, M122 and M160. Previous studies collecting *O. ishidae* from infected alders in the wild showed that insects harboured the potentially epidemic *map* genotypes M38 and M50 and have been shown to efficiently transmit them back to alders (Desqué et al., 2019).

The diversity of different genotypes seems to be greater in hazelnut than in grapevine, with more genotypes discovered in a single orchard at NE than in the entire vineyards of Slovenia. A possible reason for this could be that polyphagous vectors feeding on a variety of plants (e.g. *O. ishidae*) introduce different genotypes from outside the orchards (e.g. from alders, clematis). However, infection rates are higher for specialised vector species such as *S. titanus* in vineyards (Bressan et al., 2006) than for polyphagous vectors, which tend to feed on a variety of also non-infected plants (Lessio et al., 2016; Casati et al., 2017). The high diversity of detected *map* genotypes in the one hazelnut orchard does not support a possible transmission by planting of infected material, which could then also spread inside the orchard by root bridges (Lešnik et al., 2008). We can assume that polyphagous *O. ishidae* may be responsible for genotype diversity in hazelnut and possibly also for genotype fluxes between orchards and vineyard compartments. Other woody plant hosts present in the wild (alder or hazel) could serve as a source or relay between both compartments. Other autochthonous vectors might also be involved, as indicated by our finding of genotype M48 in hazelnuts. It was previously found in German grapevines and belongs to vectotype I, which is compatible with Macropsinae but not with Deltocephalinae (*S. titanus* and perhaps *O. ishidae*) (Malembic-Maher et al., 2020). M48 has been frequently detected in alders and in the alder Macropsinae *Oncopsis alni* in France and Germany (Malembic-Maher et al., 2020). This finding contributes to the hypothesis that alders are the original reservoir also for the genotypes found on hazelnut.

This study provides evidence that grapevine and hazelnut can harbour the same epidemic *map* genotypes of 16SrV phytoplasmas. Considering our results, the invasive *O. ishidae* is most likely the causal agent of transmission, as it was prevalent in hazelnut orchards harbouring the same genotypes as the infected plants. It could also potentially transmit these phytoplasmas to grapevines, as it has been shown to transmit 16SrV phytoplasmas from alders to grapevines at low frequency (Desqué et al., 2019). The goal of confirming *O. ishidae* as a vector between hazelnuts and testing its potential to transmit from hazelnuts to grapevines will be addressed in future research, but we should be cautious not to exclude other possible vectors. If transmission is proven, action would need to be taken against this vector, whose ecology needs further research. This will provide more clarity on how the overlapping *map* genotypes got onto the hazelnut: direct linkage from grapevine through the vector or a linkage through alder as the original reservoir. Future research will also dive into ecological studies to decipher host plants, vectors and the diversity of *map* genotypes in wild environment outside orchards and vineyards. Measuring fluxes of phytoplasma genotypes and their vectors that connect vineyards and orchards through wild environments with high throughput metabarcoding technologies will help to monitor new risks of future emergence and outbreak development.

## Data availability statement

The datasets presented in this study can be found in online repositories. The names of the repository/repositories and accession number(s) can be found in the article/**Supplementary Material**.

## Ethics statement

Ethical review and approval were not required for the study on animals in accordance with the local legislation and institutional requirements.

## Author contributions

ZZ participated in design of study, collection of material, laboratory experiments, genotyping analyses, data curation, visualisation and writing the manuscript. GS collected insect material. TJ and JB performed part of experiments in Slovenia. SP and PS helped perform MLSA experiments in France. Supervision and review were done by AV, NM, SM-M, XF. NM was included in conceptualisation and study design, formal analyses, funding acquisition and coordinated the study. All authors contributed to the article and approved the submitted version.
