# Corrigendum: Epidemiology of flavescence dorée and hazelnut decline in Slovenia: geographical distribution and genetic diversity of the associated 16SrV phytoplasmas

**DOI:** 10.3389/fpls.2023.1261658

**Published:** 2023-08-31

**Authors:** Zala Kogej Zwitter, Gabrijel Seljak, Tjaša Jakomin, Jakob Brodarič, Ana Vučurović, Sandra Pedemay, Pascal Salar, Sylvie Malembic-Maher, Xavier Foissac, Nataša Mehle

**Affiliations:** ^1^ National Institute of Biology, Department of Biotechnology and Systems Biology, Ljubljana, Slovenia; ^2^ Sensor Technologies, Jožef Stefan International Postgraduate School, Ljubljana, Slovenia; ^3^ National Research Institute for Agriculture, Food and Environment, Fruit Biology and Pathology, Bordeaux, France; ^4^ School for Viticulture and Enology, University of Nova Gorica, Nova Gorica, Slovenia

**Keywords:** epidemiology, plant disease, vectotype, *Vitis vinifera*, *Corylus avellana*

In the published article, there was an error in **Table 2** as published. There were mistakes in listed references to GenBank accession numbers: for AM384887, AM384886, and AM384893, the correct reference is (Arnaud et al., 2007) and not (Malembic-Maher et al., 2020); for LN850369, the correct reference is (Elek et al., unpublished) and not (Malembic-Maher et al., 2020); and for FN811141, the correct reference is (Malembic-Maher et al., 2011) and not (Malembic-Maher et al., 2020). The wrong accession number was provided for genotype M122, which should be LN850372 and not LT222007 with reference (Elek et al., unpublished) and not (Plavec et al., 2019). The corrected **Table 2** and its caption “Summary of discovered *map* genotypes from 2017 to 2022 in different hosts” appear below.

**Table 2:** Summary of discovered *map* genotypes from 2017 to 2022 in different hosts.

Map-FD cluster	Genotype	GenBank acc no.	Reference	Host	Number of samples
FD1	M50	AM384887	(Arnaud et al., 2007)	Grapevine	4
				Hazelnut	8*
				*O. ishidae*	1
	M158	OX417123**	This study	Grapevine	7
	M159	OX417124**	This study	Hazelnut	1
	M160	OX417125**	This study	Hazelnut	1
				*O. ishidae*	1
	M161	OX417126**	this study	Hazelnut	1*
FD2	M54	AM384886	(Arnaud et al., 2007)	Grapevine	147
				*S. titanus*	5
	M38	LN850369	(Elek et al., unpublished)	Grapevine	11
				Hazelnut	12*
				*O. ishidae*	5
	M122	LN850372	(Elek et al., unpublished)	Grapevine	2
				Hazelnut	2*
				*O. ishidae*	2
	M162	OX417127**	This study	Hazelnut	3*
FD3	M51	FN811141	(Malembic-Maher et al., 2011)	Grapevine	5
Grapevine non-epidemic isolate	M48	AM384893	(Arnaud et al., 2007)	Hazelnut	2*

* In 11 samples, roots and shoots were analyzed separately (counted as one sample here; results of separate analysis are shown in **Supplementary Table 3** ).

** Sequences were deposited to the European Nucleotide Archive (ENA) under https://www.ebi.ac.uk/ena/browser/view/OX417123-OX417147.

In the published article, there was an error in **Supplementary Table 2**. There were missing accession numbers for isolates D1385/19, D1448/20, D1455/20, SB21-5-P, and SB21-10-K for genes *map, vmpA-R1, dnaK, tuf, rplV*, and *rplF*. The new caption of the **Supplementary Table 2** is “GenBank accession number list of *map, vmpA-R1, dnaK, tuf, rplV*, and *rplF* genes used in phylogenic studies of grapevine and hazelnut isolates of 16SrV phytoplasma in Slovenia (accession numbers of *tuf, rplV*, and *rplF* for other isolates are published in Malembic-Maher et al., 2011)”.

The authors apologize for these errors and state that this does not change the scientific conclusions of the article in any way. The original article has been updated.

