# Supplementary material for: Epidemiology of flavescence dorée and hazelnut decline in Slovenia: geographical distribution and genetic diversity of the associated 16SrV phytoplasmas
Source: Front Plant Sci. 2023 Jul 4;14:1217425. doi: 10.3389/fpls.2023.1217425 (PMC10352807; doi:10.3389/fpls.2023.1217425)
Supplement: Supplementary file 1 [file DataSheet_1.zip › Data Sheet 1 (41)/Supplementary data/Supplementary_table_1_and_captions.docx]

Supplementary Material

Epidemiology of Flavescence dorée and hazelnut decline in Slovenia: geographical distribution and genetic diversity of the associated 16SrV phytoplasmas

Zala Kogej Zwitter^*^, Gabrijel Seljak, Tjaša Jakomin, Jakob Brodarič, Ana Vučurović, Sandra Pedemay, Pascal Salar, Sylvie Malembic-Maher, Xavier Foissac, Nataša Mehle

*** Correspondence:** Zala Kogej Zwitter: zala.kogej.zwitter@nib.si

# Supplementary Tables

**Supplementary table 1.** List of all nested PCR (nPCR) tests used in this study for additional new isolate characterisation of newly discovered *map* genotypes in grapevine and hazelnut in Slovenia.

| Gene locus and use | Primer name | Primers (5’ -3’) | Cycling conditions | Reference |
| --- | --- | --- | --- | --- |
| *tuf* PCR | fusA2tuf-F0 | GCTAGATATGCTAAAACGCC | 94°C, 1’; (94°C, 30’’; 55°C, 30’’; 66°C, 1’) x 35; 66°C, 5’ | (Malembic-Maher et al., 2011), here adapted with fusA2tuf-F0 |
|  | FDTUF-R1 | GTTCTTCCGCCTTCACGTAC |  |  |
| *tuf* nPCR and sequencing | FDTUF-F1 | ATTGGTCATGTAGACCATGG |  |  |
|  | FDTUF-R2 | CTTGTTCCTTCTTCGATCGC |  |  |
| *rplV* PCR | RpVF1 | TCGCGGTCATGCAAAAGGYG | 94°C, 1’; (94°C, 1’; 55°C, 1’; 66°C, 1’30’’) x 35; 66°C, 5’ | (Malembic-Maher et al., 2011) |
|  | RpVR1 | ACGATATTTAGTYYTTTTTGG |  |  |
| *rplV* nPCR and sequencing | RpVF1A | AGGYGATAAAAAAGTTTCAAAA |  |  |
|  | RpVR1A | GGCATTAACATAATATATTATG |  |  |
| *rplF* PCR | GRV-S8-F1 | ACTTCAAAGGGTATTTTGAC | 94°C, 1’; (94°C, 30’’; 55°C, 30’’; 66°C, 1’) x 35; 66°C, 5’ | (Malembic-Maher et al., 2011), here adapted with GRV-L18-R0 |
|  | GRV-L18-R0 | CCTCTTCTCTTGCAACATTCGC |  |  |
| *rplF* nPCR and sequencing | GRV-S8-F2 | ATAGGTGGAGAAGTTTTAGC |  |  |
|  | GRV-L18-R2 | CCATCACGATCAAAAACAAC |  |  |
| *dnaK* PCR | dnaK-F2 | CACCTTCAATTGTAGCATTCAG | 94°C, 1’; (94°C, 1’; 55°C, 1’; 66°C, 1’30’’) x 35; 66°C, 5’ | This study |
|  | dnaK-R3 | TCTTCTGATAAAGAACCGCTTCC |  |  |
| *dnaK* nPCR and sequencing | dnaK-F | TTAGGCGGAGGAACTTTCGAC | 94°C, 1’; (94°C, 30’’; 55°C, 30’’; 66°C, 30’’) x 35; 66°C, 5’ | (Rossi et al., 2019) |
|  | dnaK-R | AAGCTCCCATCGCAACTACT |  |  |
| *vmpA-R1* PCR | VMPA-F5 | CCTTATCAACTGGATATGGT | 94°C, 5’; (94°C, 30’’; 55°C, 30’’; 66°C, 2’30’’) x 35; 66°C, 5’ | (Malembic-Maher et al., 2020) and (Rossi et al., 2019) |
|  | VMPA-R3 | CTGATGCGTTTAGCCACTTC |  |  |
| *vmpA-R1* nPCR | VMPA-F8 | TTATAGAAATTATTCTCACAA | 94°C, 1’; (94°C, 30’’; 55°C, 30’’; 66°C, 30’’) x 35; 66°C, 5’ |  |
|  | VMPA-R9 | TAAAAMAGTMGATAATTCAAC |  |  |
| *vmpA-R1* sequencing | VMPA-F3 | GATAGGAAAACAAAATGATAG |  |  |
|  | VMPA-F8 | Same as nPCR |  |  |

**Supplementary table 2.** GeneBank accession number list of *map*, *dnaK* and *vmpA* genes used in phylogenic studies of grapevine and hazelnut isolates of 16SrV phytoplasma in Slovenia

**Supplementary table 3.** Table of all grapevine, hazelnut and leafhoppers samples, for which *map* genotypes were determined in this study

# Supplementary Figures

**Supplementary Figure 1.** The percentage of detected *map* genotypes in grapevine from 2017 to 2021 in Slovenia.

**Supplementary figure 2.** The percentage of detected *map* genotypes in hazelnut from 2017 to 2021 in Slovenia.
