# Supplementary figures and images for: Epidemiology of flavescence dorée and hazelnut decline in Slovenia: geographical distribution and genetic diversity of the associated 16SrV phytoplasmas
Source: Front Plant Sci. 2023 Jul 4;14:1217425. doi: 10.3389/fpls.2023.1217425 (PMC10352807; doi:10.3389/fpls.2023.1217425)

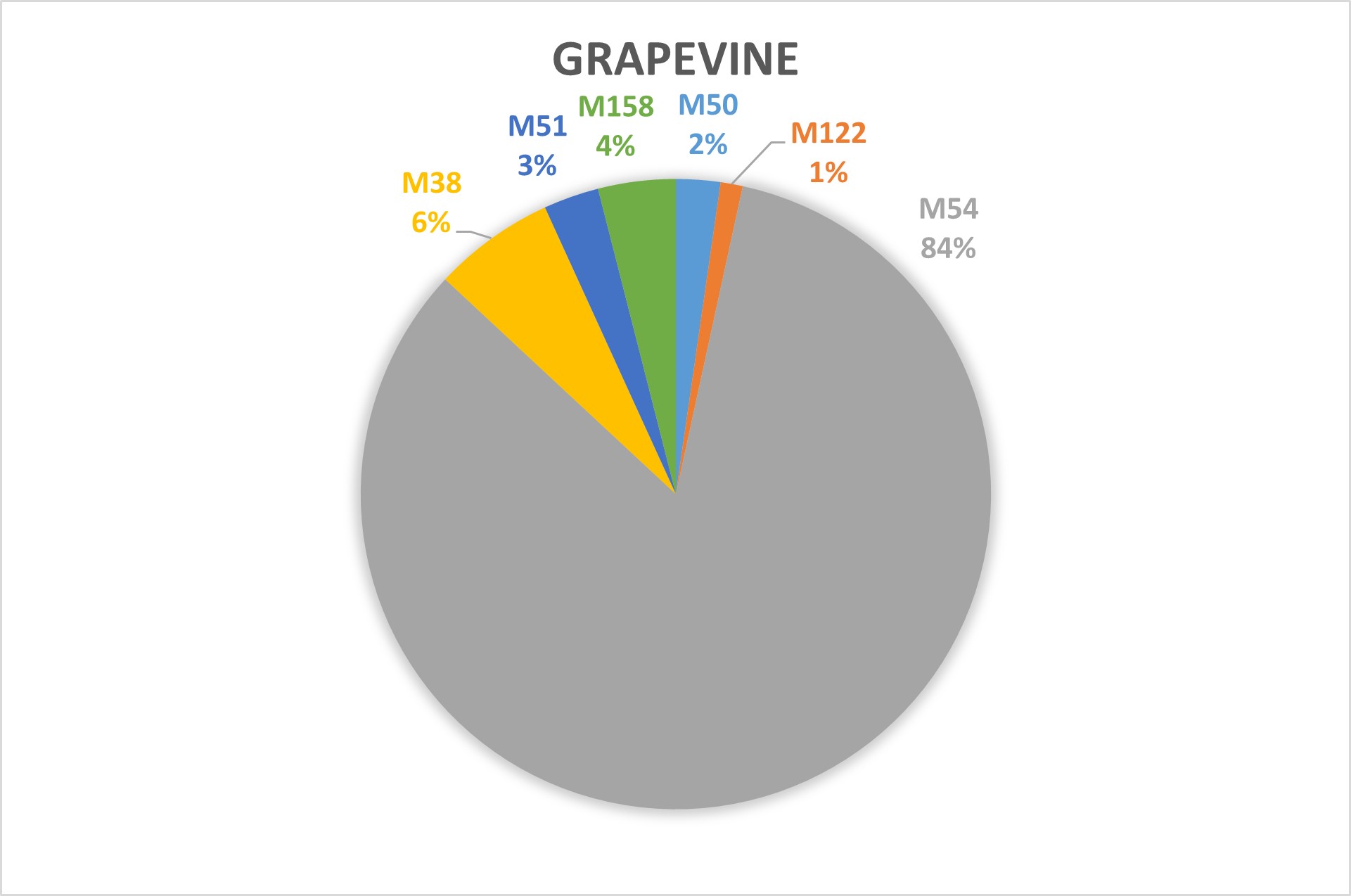

Supplement: Supplementary file 1 [file DataSheet_1.zip › Data Sheet 1 (41)/Supplementary data/Supplementary_figure_1.jpg]

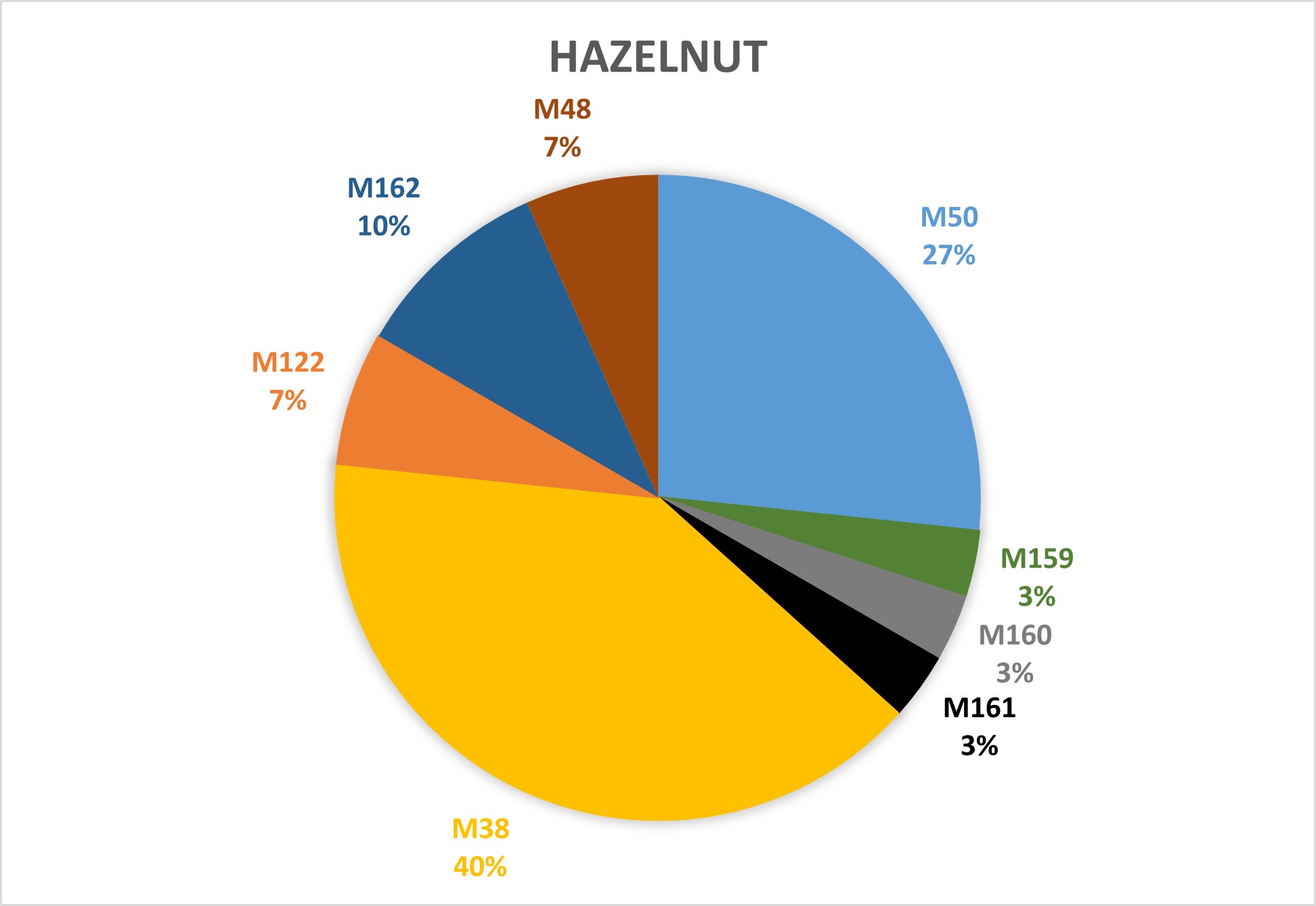

Supplement: Supplementary file 1 [file DataSheet_1.zip › Data Sheet 1 (41)/Supplementary data/Supplementary_figure_2.jpg]
